# An unusual rash heralding pleomorphic transformation of mantle cell lymphoma

**DOI:** 10.1002/jha2.64

**Published:** 2020-09-10

**Authors:** Subbramanian Palaniappan, Gavin Laing, Alexandra Bonsall, Dominic Culligan

**Affiliations:** ^1^ Haematology Department Aberdeen Royal Infirmary, NHS Grampian Aberdeen UK; ^2^ University of Aberdeen Aberdeen UK; ^3^ Pathology Department Aberdeen Royal Infirmary, NHS Grampian Aberdeen UK; ^4^ Dermatology Department Aberdeen Royal Infirmary, NHS Grampian Aberdeen UK

A 75‐year‐old male taking ibrutinib for 5 months for relapsed mantle cell lymphoma (MCL) presented with rising lymphocyte count, headache, peri‐orbital numbness, and a rapidly progressive rash. Large areas of skin at the chest and back were indurated and affected by confluent erythema (Figure A). The edges of the rash were plaque‐like in nature with peripheral maculopapular lesions. A mixture of indurated erythema and maculopapular rash was also present at the abdomen, shoulders, extensor surface of the arms, face, and scalp. Punch skin biopsy (3 mm × 5 mm) from the upper arm demonstrated a perivascular and periadnexal lymphoid infiltrate of atypical, pleomorphic, lymphoid cells in the superficial dermis and focally extending into deep dermis (Figure B). Immunohistochemistry was positive for CD20, CD79a, PAX5, and BCL2. Cells also showed positive, but weak, expression of CD5. The Ki‐67 proliferation index was very high (80–90%, Figure C), compared with 10–15% in previous lymph node biopsies. Interestingly, cyclin D1 expression, which was strongly positive at presentation with nodal MCL in 2014, was now negative, though fluorescent in situ hybridisation (FISH) testing on 55 interphase cells from the skin biopsy confirmed *IGH/CCND1* rearrangement. The diagnosis was high‐grade transformation of MCL, pleomorphic variant. Along with blastoid variant, this represents aggressive progression of MCL. The high Ki‐67 index is prognostically important independent of morphology. Staging confirmed involvement of cerebrospinal fluid, bone marrow, lymph nodes, and spleen.


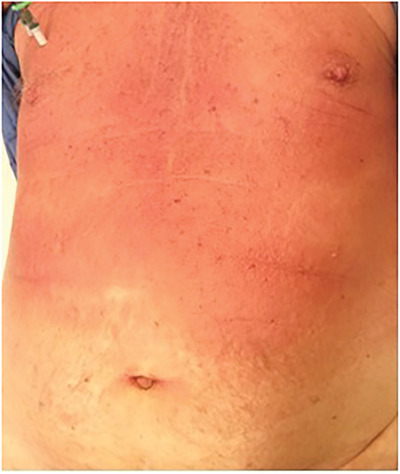


Figure A


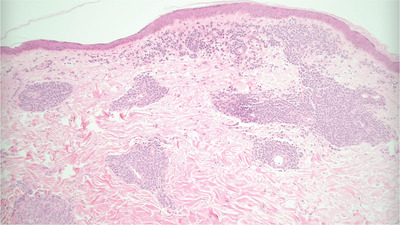


Figure B


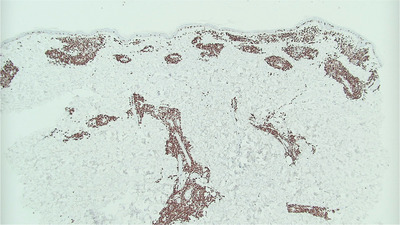


Figure C

Skin involvement with B‐cell lymphomas, especially MCL, is less frequent than with T‐cell lymphomas and commonly manifests as isolated nodules. This confluent and indurated rash was unusual and heralded extensive extranodal pleomorphic transformation of MCL.

